# Assessment of geographic access to monoclonal antibodies in the United States

**DOI:** 10.1093/jtm/taac018

**Published:** 2022-03-04

**Authors:** Benjamin Rader, Christopher M Whaley, Wesley S Rogers, Professor John S Brownstein, Jonathan Cantor

**Affiliations:** Computational Epidemiology Lab, Boston Children’s Hospital, Boston, MA, USA; Department of Epidemiology, Boston University School of Public Health, Boston, MA, USA; RAND Corporation, Santa Monica, CA, USA; NewYork- Presbyterian Hospital - Weill Cornell Medical Center, New York, NY, USA; Computational Epidemiology Lab, Boston Children’s Hospital, Boston, MA, USA; Harvard Medical School, Havard University, Boston, MA, USA; RAND Corporation, Santa Monica, CA, USA

**Keywords:** monoclonal antibodies, monoclonal antibody, access to treatment, covid-19 treatment, access to covid-19 care, antibody treatment, access to care, SARS-CoV-2 treatment, COVID-19 treatment disparities

## Abstract

Using spatial modeling techniques, we highlight disparities in access to monoclonal antibodies (mAbs) used to treat COVID-19 patients. Fifteen million individuals in the United States must travel over 30 min to access mAbs. Areas with lower COVID-19 vaccination rates have worse access to essential COVID-19 therapeutics.

Neutralizing monoclonal antibodies (mAbs) reduce viral loads in mild to moderate COVID-19 patients^1^ and may prevent admission or death in non-hospitalized patients according to the United States (U.S.) Department of Health and Human Services (HHS).^2^ However, the production of mAbs continues to lag behind daily COVID-19 cases and has led to national shortages and forced rationing.^1^^,^^3^ Concerns also exist that rural facilities may have reduced access to these treatments because they lack the capacity for large-scale infusion operations.^1^ In September 2021, the Department of Health and Human Services (HHS) announced a new mAb distribution method due to a drastic uptick in COVID-19 cases. HHS calculates incident and hospitalized cases and allocates each state mAbs accordingly, adjusting each week’s supply based on previously unused allocations.^2^ It is reported that existing measures of local disease burden may be an ineffective marker for the distribution of mAbs.^1^ In addition, only certain mAbs are an effective form of treatment for the omicron variant of COVID-19.^4^ Although socio-geographic disparities in accessing COVID-19 resources (e.g. testing,^5^ vaccinations^6^) have been well documented and recent reports suggest disparities among mAb recipients,^7^ to our knowledge there has been no examination of how the current distribution scheme affects geographic access to mAbs across the United States.

We used previously described methods^6^ to measure the travel time from each populated census tract in the contiguous U.S. (*n* = 71 927) to the closest facility that had received a delivery of mAbs (*n* = 4240, HHS Data Hub, accessed 6 January 2022) and the closest facility administering a COVID-19 vaccine (*n* = 36 259, Vaccines.gov,^5^ accessed 6 January 2022). We compared the travel time to a vaccine and mAbs to understand the tradeoff in receiving preventive COVID-19 care as opposed to therapeutic COVID-19 care. Travel times were computed by estimating the quickest means of transversal (e.g. car, public transportation) from each 1 km^2^ grid-square of the U.S. to the closest location of interest and averaged across all grid-squares in each tract.^8^ Extended travel times (≥30 min) were defined by the Veterans Administration’s primary care standard.^9^ We also report tract-level 2019 American Community Survey demographics, tract-level collapsed Rural–Urban Commuting Area codes,^10^ county-level COVID-19 vaccination (Covid.CDC.gov, accessed 6 January 2022), and county-level 2020 Presidential voting (ElectionLab.MIT.edu).

Individuals in the mean census tract had to travel twice as long to the nearest mAbs administration site [10.5 min (SD: 14.3)] compared with the nearest COVID-19 vaccine location [4.6 min (SD: 8.9)], although there was high variability especially across the urban–rural spectrum. In addition, a large number of individuals (*n* = 15 252 601) live in census tracts with extended travel times to mAbs, but not the COVID-19 vaccine (Figure 1). Extended travel times to mAbs were prevalent in the Mountain division of the U.S. When comparing census tracts under 30 min from mAbs/vaccines to those with extended travel times to mAbs (Table 1), the latter are on average more rural, older (4.3 years), have lower household incomes ($6389 less), higher percent white (14.5% more), more likely uninsured (0.8% more) and have poorer internet access (6.1% lower). Low-access census tracts were also 16.3 points less likely to be in majority-vaccinated counties and were 45.0 points more likely to have voted for Donald Trump in 2020.

**Figure 1:**
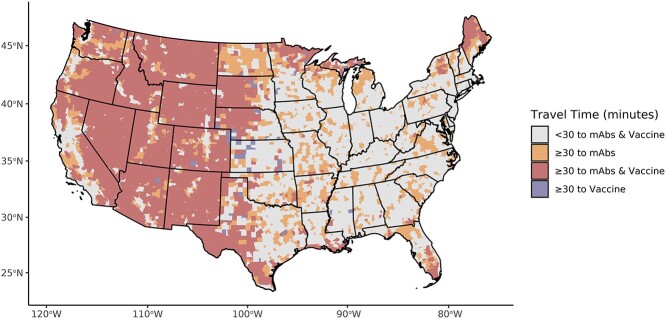
US travel times to monoclonal antibodies and the COVID-19 vaccine. Mean travel times for each census tract in the contiguous United States to the nearest location that previously (as of 6 January 2022) received a delivery of neutralizing mAbs according to the US Department of Health and Human Services and the nearest location distributing the COVID-19 vaccine (as of 6 January 2022) according to Vaccines.gov. Travel times are aggregated at the census tract level which may obscure hyperlocal access measurements, especially in large tracts like those found in the western US.

**Table 1:** Characteristics of US census tracts (*n* = 71 927) by travel time to mAbs and the COVID-19 vaccine

	<30 min to mAbs and vaccine (*n* = 66 781)	≥30 min to mAbs (*n* = 3840)	≥30 min to mAbs and vaccine (*n* = 1235)	≥30 min to vaccine (*n* = 71)	Total (*n* = 71 927)
Rural–Urban designation, *n* (%)					
Urban	51 100 (99.14%)	398 (0.77%)	43 (0.08%)	0 (0.00%)	51 541 (71.66%)
Suburban	6570 (83.38%)	1067 (13.54%)	238 (3.02%)	5 (0.06%)	7880 (10.96%)
Large rural	5259 (85.04%)	738 (11.93%)	177 (2.86%)	10 (0.16%)	6184 (8.60%)
Small rural	2205 (70.54%)	722 (23.10%)	186 (5.95%)	13 (0.42%)	3126 (4.35%)
Isolated rural	1494 (49.65%)	894 (29.71%)	579 (19.24%)	42 (1.40%)	3009 (4.18%)
No designation	153 (81.82%)	21 (11.23%)	12 (6.42%)	1 (0.53%)	187 (0.26%)
Sex					
Female, mean % (SD)	50.85 (±4.73)	49.74 (±4.98)	48.54 (±5.64)	50.14 (±2.38)	50.75 (±4.77)
Median age, years					
Mean (SD)	39.28 (±7.71)	43.57 (±7.72)	45.18 (±9.40)	43.85 (±6.76)	39.62 (±7.84)
Missing, *n* (%)	23 (0.03%)	1 (0.03%)	0 (0.00%)	0 (0.00%)	24 (0.03%)
Household income, $1000					
Mean (SD)	33.70 (±14.16)	27.31 (±6.98)	26.58 (±7.05)	26.58 (±5.26)	33.23 (±13.88)
Missing, *n* (%)	77 (0.12%)	5 (0.13%)	5 (0.40%)	0 (0.00%)	87 (0.12%)
Race, mean % (SD)					
White	71.35 (±25.22)	85.82 (±16.76)	81.35 (±25.67)	81.06 (±26.75)	72.31 (±25.09)
Black	14.55 (±22.18)	7.09 (±14.45)	2.87 (±10.12)	7.80 (±20.79)	13.95 (±21.80)
Hispanic	17.21 (±21.72)	9.35 (±16.32)	14.47 (±20.34)	11.59 (±18.24)	16.74 (±21.52)
American Indian or Alaska Native^a^	0.69 (±2.70)	1.66 (±6.95)	9.71 (±24.07)	6.71 (±19.50)	0.90 (±4.59)
Asian	5.18 (±9.18)	0.79 (±1.39)	0.66 (±1.43)	0.48 (±0.64)	4.86 (±8.93)
Hawaiian or Pacific Islander^a^	0.13 (±0.60)	0.08 (±0.37)	0.12 (±0.59)	0.07 (±0.20)	0.13 (±0.59)
Two or more races	3.20 (±2.78)	2.43 (±2.49)	2.55 (±2.73)	2.19 (±2.15)	3.15 (±2.77)
Other race	4.90 (±8.89)	2.13 (±5.46)	2.73 (±6.58)	1.70 (±5.20)	4.71 (±8.73)
Health insurance, mean % (SD)					
Employer	45.65 (±15.03)	39.00 (±10.72)	33.68 (±12.52)	36.18 (±8.79)	45.08 (±14.94)
Direct	6.51 (±4.22)	6.68 (±3.87)	7.72 (±5.48)	8.32 (±4.64)	6.54 (±4.23)
Medicare	5.36 (±3.13)	6.78 (±2.99)	7.70 (±3.66)	6.29 (±2.33)	5.48 (±3.16)
Medicaid	15.81 (±12.28)	16.03 (±8.52)	17.01 (±11.44)	16.10 (±10.54)	15.84 (±12.09)
Veterans administration	0.28 (±0.51)	0.37 (±0.52)	0.42 (±0.57)	0.33 (±0.31)	0.29 (±0.51)
Tricare	0.96 (±3.86)	0.79 (±2.29)	0.88 (±3.86)	0.48 (±0.83)	0.95 (±3.79)
Two or more	5.96 (±3.25)	6.61 (±2.86)	6.01 (±3.34)	6.53 (±2.64)	5.99 (±3.24)
Uninsured	8.75 (±7.02)	9.50 (±6.04)	11.34 (±7.42)	10.20 (±6.27)	8.84 (±6.98)
Missing, *n* (%)	96 (0.14%)	6 (0.16%)	2 (0.16%)	0 (0.00%)	104 (0.14%)
No internet					
Mean (SD)	14.73 (±10.36)	20.86 (±9.76)	23.58 (±14.09)	25.78 (±11.15)	15.22 (±10.56)
Missing, *n* (%)	167 (0.25%)	8 (0.21%)	4 (0.32%)	0 (0.00%)	179 (0.25%)
Majority of adults fully vaccinated^b^, % (95% CI)	92.73 (92.53–92.93)	76.43 (75.05–77.76)	76.36 (73.87–78.68)	76.06 (64.20–85.05)	91.56 (91.36–91.76)
Won by Donald Trump in 2020^b^, N (95% CI)					
Mean (SD)	37.47 (37.10–37.84)	82.50 (81.25–83.68)	75.38 (72.86–77.74)	85.92 (75.16–92.68)	40.57 (40.22–40.93)
Missing, *n* (%)	0 (0.00%)	0 (0.00%)	3 (0.24%)	0 (0.00%)	3 (0.00%)

^a^Only contiguous United States included in analysis.

^b^County-level statistic (e.g. 40.57% of census tracts are in a county won by Trump and 91.56% of census tracts are in a county where the majority (50%+) of adults (18+) are fully vaccinated against COVID-19)

In this study, we quantified access to two critical pandemic resources and show that travel times to mAbs are double travel times to the COVID-19 vaccine. This result highlights the relative tradeoff and additional cost of receiving mAbs compared with the vaccine. The travel cost is on top of the significant financial and administrative burden of using mAbs,^1^ as opposed to a COVID-19 vaccine. The complex logistics of direct administration of mABs (e.g. testing requirements, trained administrators) have resulted in a distribution scheme that has left over 15 million people in the U.S. with extended travel times to this COVID-19 therapy. Our results also highlight profound disparities in access to mAbs and that the areas that are most susceptible to COVID-19 due to low vaccination rates are also less likely to have convenient access to this treatment. These communities also have lower household income and less access to the internet which may exacerbate health service access barriers.

There are important limitations to this study. Our sociodemographic measures are population aggregates, and our measure of travel time assumes equal access to the quickest means of transportation. Here we use mAB delivery as a proxy for mAB administration which may obscure its complex distribution patterns. Further research should leverage individual-level mAb recipient data to quantify how geographic access differences manifests in outcome disparities. However, this study is the first to use national data to quantify the geographic access of mAbs. Our results show that the urban–rural barriers that are common with accessing health services^10^ and other COVID-19 resources^5^^,^^6^ are also present in accessing mAbs.

Shifting COVID-19 variants and their respective treatments suggest a dynamic landscape where various therapies (e.g. mAB formula, direct acting antivirals, etc.) may fluctuate in appropriateness. Comprehensive solutions including unconventional mAB delivery technologies such as home administration (e.g. via paramedics) and local community clinic partnerships are essential to remedy access barriers and to ensure equitable availability of this important COVID-19 treatment.

## Funding

This work was supported by Google.org via the Tides Foundation [TF2003-089662 to J.S.B.] and the National Institute on Aging [K01AG061274 to C.M.W.].

## Conflicts of Interest

The authors have no conflicts of interest to declare.

## Data Sharing

All data used in this analysis is accessible from publicly available sources.

## Author Contributions

Rader, Whaley, Brownstein and Cantor conceived of the study. All authors were involved in the acquisition, analysis, or interpretation of data. Rader and Cantor wrote the original draft of the manuscript. All authors engaged in critical revision of the manuscript for important intellectual content. Whaley and Brownstein obtained funding and supervised the project. All authors have seen and approve the final text.
